# Chronic recurrent dehydration associated with periodic water intake exacerbates hypertension and promotes renal damage in male spontaneously hypertensive rats

**DOI:** 10.1038/srep33855

**Published:** 2016-09-22

**Authors:** Lucinda M. Hilliard, Katrina M. Mirabito Colafella, Louise L. Bulmer, Victor G. Puelles, Reetu R. Singh, Connie P. C. Ow, Tracey Gaspari, Grant R. Drummond, Roger G. Evans, Antony Vinh, Kate M. Denton

**Affiliations:** 1 Cardiovascular Disease Program, Biomedicine Discovery Institute and Department of Physiology, Monash University, Melbourne, Victoria, 3800, Australia; 2 Department of Anatomy and Developmental Biology, Monash University, Melbourne, Victoria, 3800, Australia; 3 Department of Pharmacology, Monash University, Melbourne, Victoria, 3800 Australia

## Abstract

Epidemiological evidence links recurrent dehydration associated with periodic water intake with chronic kidney disease (CKD). However, minimal attention has been paid to the long-term impact of periodic water intake on the progression of CKD and underlying mechanisms involved. Therefore we investigated the chronic effects of recurrent dehydration associated with periodic water restriction on arterial pressure and kidney function and morphology in male spontaneously hypertensive rats (SHR). Arterial pressure increased and glomerular filtration rate decreased in water-restricted SHR. This was observed in association with cyclic changes in urine osmolarity, indicative of recurrent dehydration. Additionally, water-restricted SHR demonstrated greater renal fibrosis and an imbalance in favour of pro-inflammatory cytokine-producing renal T cells compared to their control counterparts. Furthermore, urinary NGAL levels were greater in water-restricted than control SHR. Taken together, our results provide significant evidence that recurrent dehydration associated with chronic periodic drinking hastens the progression of CKD and hypertension, and suggest a potential role for repetitive bouts of acute renal injury driving renal inflammatory processes in this setting. Further studies are required to elucidate the specific pathways that drive the progression of recurrent dehydration-induced kidney disease.

Water is essential for life. Increasing epidemiological evidence suggests irregular water intake is a risk factor for the development and progression of chronic kidney disease (CKD). Several observational studies have documented an inverse relationship between progression of CKD and fluid intake or urine volume^1^,^2^,^3^. Furthermore, it has been demonstrated that high urine volume correlates with a reduced risk of primary and secondary nephrolithiasis^4^,^5^,^6^. Recurrent dehydration associated with irregular water intake and occupational heat stress has been linked to an epidemic of CKD in hot coastal communities of Central America^7^,^8^. Some evidence also exists that low urine flow favours the development of hypertension; a major risk factor for the development and progression of CKD^9^. Moreover, there is evidence from studies in 5/6 nephrectomized rats that increased water consumption slows the progression of CKD^10^.

It is well recognized that the kidney is the major organ responsible for the regulation of whole-body fluid homeostasis. However, although the underlying mechanisms that contribute to the renal regulation of body fluid homeostasis have been extensively studied, the direct consequences of periodic water intake on the progression of CKD and associated underlying mechanistic pathways have not been thoroughly investigated. Such investigations in this field are long overdue considering CKD is commonly asymptomatic until its advanced form and, consequently, often remains undiagnosed. Moreover, numerous population-based studies have revealed that around 10–11% of the adult population demonstrate some degree of renal dysfunction^11^. Certainly, an improved understanding of the impact of recurrent dehydration on the progression of CKD and mechanisms that drive recurrent dehydration-induced kidney disease could lead to the generation of improved evidence-based water intake guidelines for the general population, as well as the identification of novel therapeutic strategies that protect against the progressive course of CKD.

To address this knowledge gap, the aim of the current study was to investigate the impact of periodic water restriction on arterial pressure and kidney function and structure in male spontaneously hypertensive rats (SHR). This was achieved by restricting drinking in SHR to a 2-hour period each day for 4 weeks. The SHR was selected for this study since it is a well-established genetic model of essential hypertension that demonstrates a gradual decline in renal function and progression of CKD with age. Our study commenced in SHR at 12 weeks of age prior to any evidence of hypertensive kidney damage, which is morphologically evident from approximately 30 weeks of age^12^,^13^,^14^. There is a strong association between T cell infiltrate, hypertension and renal dysfunction, although the precise mechanisms remain to be determined. We, and others, have identified an accumulation of T cells with a pro-inflammatory phenotype in mouse kidneys, which is associated with changes in blood pressure^15^,^16^,^17^. Therefore, we investigated the impact of recurrent dehydration associated with periodic water restriction in 12-week old SHR to represent a population at risk of CKD. We hypothesized that recurrent dehydration associated with periodic water intake exacerbates the progression of CKD and hypertension by recruiting T-cells and promoting a pro-inflammatory environment in the kidney.

## Results

### Daily water intake

**Figure 1:**
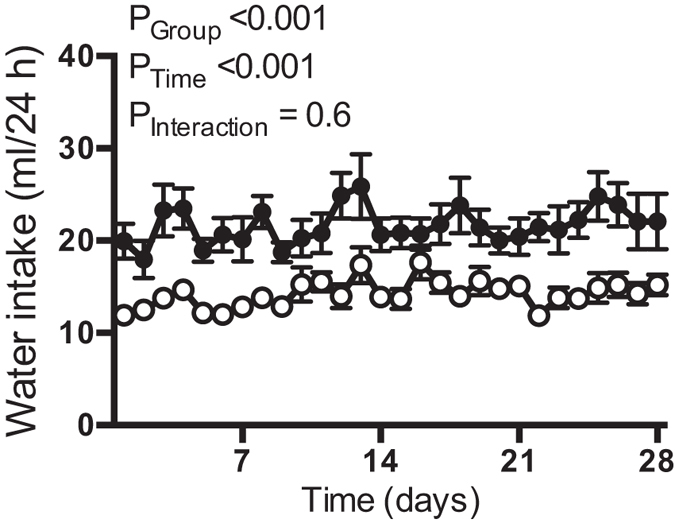
Daily water intake: Daily water intake for control (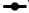) and water-restricted SHR (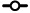) during the 4-week water restriction study. All data are presented as mean ± SEM. Data were analyzed using repeated-measures ANOVA. No correction was made for sphericity. n = 8–13 per group.

Daily water consumption was on average ~34% less in the water-restricted SHR than the control SHR (Fig. 1).

### Body and kidney weight

Body weight did not differ significantly between the control and water-restricted SHR at baseline. Over time, body weight increased by 31 ± 2% in control and 26 ± 2% in water-restricted SHR. However, at the end of the water-restriction protocol body weight measured at the end of the dehydration period prior to replenishment was ~6% less in the water-restricted than control SHR (P < 0.05) (see Supplementary Fig. S1).

Kidney weight was not significantly different between the control and water-restricted SHR groups. However, kidney to body weight ratio was ~8% greater in water-restricted SHR than control SHR (P < 0.001). (see Supplementary Fig. S1).

### Mean arterial pressure, heart rate and locomotor activity

**Figure 2:**
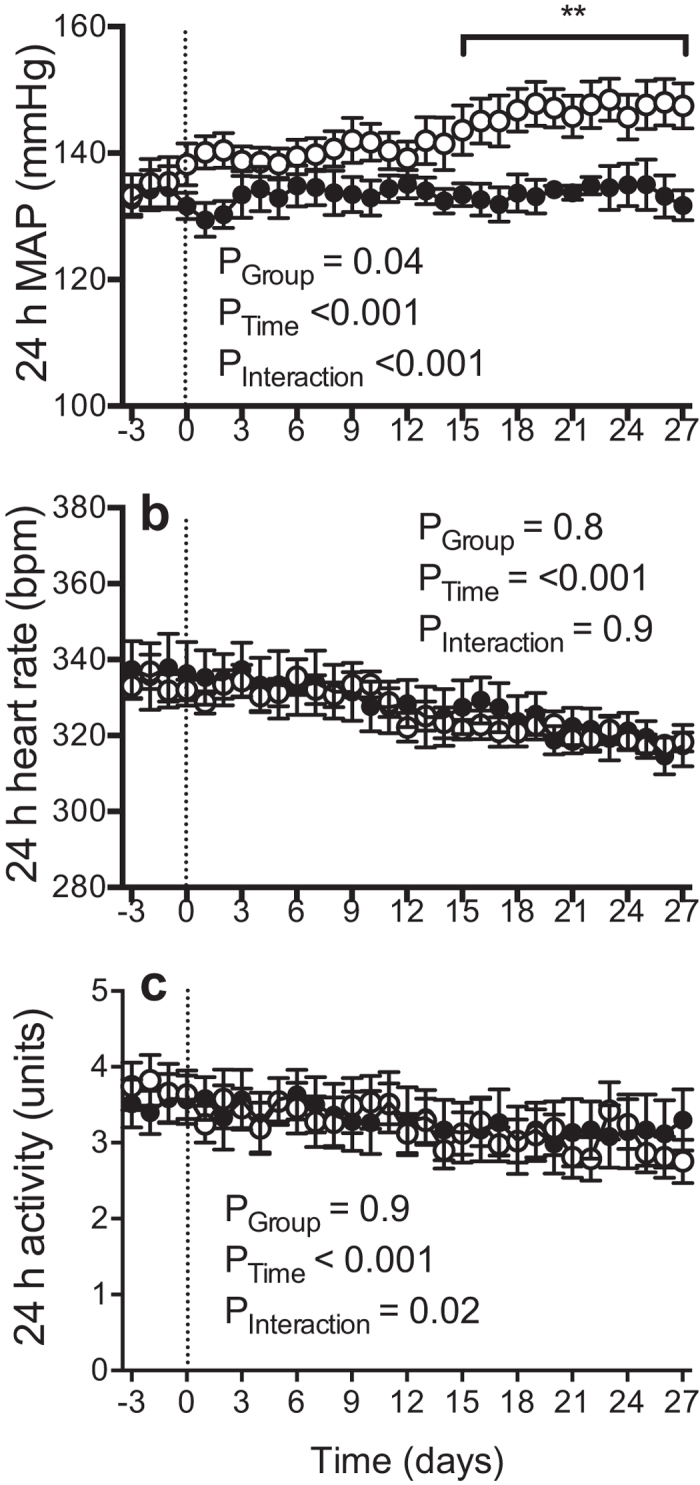
24 h mean arterial pressure, heart rate and locomotor activity across the 4-week water restriction protocol: **(a)** 24-hour average mean arterial pressure (MAP), **(b)** heart rate and **(c)** locomotor activity in control (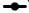) and water-restricted (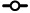) SHR at baseline and across the 4-week protocol of water restriction. All data are presented as mean ± SEM. Data were analyzed using repeated-measures ANOVA with Bonferroni’s post-hoc tests (28 comparisons). No correction was made for sphericity. **P ≤ 0.01 versus baseline (3-day average). n = 6–9 per group.

**Figure 3:**
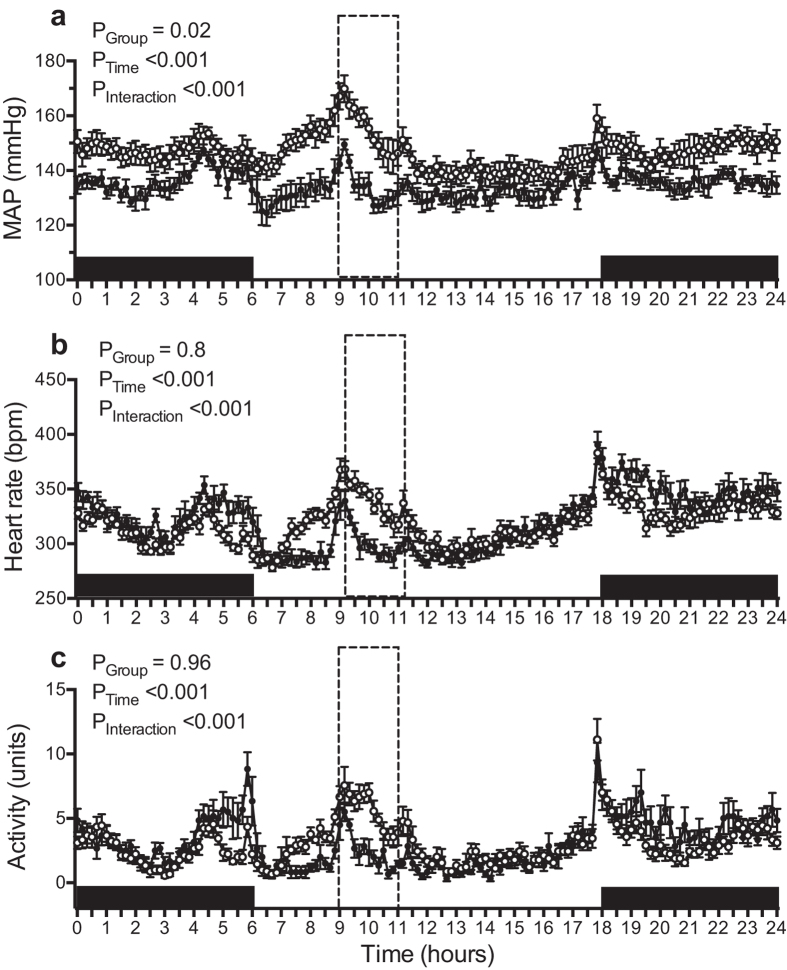
Hourly mean arterial pressure, heart rate and locomotor activity during the final week of the water restriction protocol: (**a**) Average hourly mean arterial pressure (MAP), (**b**) heart rate, and (**c**) locomotor activity during the final week of the experimental protocol in control (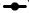) and water-restricted (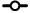) SHR. All data are presented as mean ± SEM. Data were analyzed using repeated-measures ANOVA using the factors group, time and the interaction between group and time. n = 6–9 per group.

Baseline MAP was not significantly different between the control and water-restricted SHR. In the control SHR, MAP remained close to baseline throughout the duration of the experiment (Fig. 2a). In contrast, MAP progressively increased over time in the water-restricted SHR, to be ~13 mmHg greater at the end of the protocol than at baseline. This increase in arterial pressure was evident from day 15 of the water restriction protocol. Furthermore, examination of hourly MAP averages across the final week of the water restriction protocol confirmed that MAP was consistently greater in water-restricted than control SHR across the entire 24-hour period (Fig. 3a). This was particularly evident immediately prior to and during the water access period where arterial pressure spiked in both treatment groups but to a greater extent in the water-restricted SHR. The circadian pattern of MAP was not significantly affected by water restriction although there was a trend for a lower day-night difference in water-restricted than control SHR (P = 0.08). The day-night difference in MAP was 5 ± 1 mmHg and 4 ± 1 mmHg during baseline in the control and water-restricted SHR, respectively. The average day-night difference in MAP during the final week of the water-restriction protocol was 5 ± 2 mmHg and 2 ± 1 mmHg in the control and water-restricted SHR, respectively.

Heart rate was also similar between the control and water-restricted SHR at baseline. Mean heart rate decreased similarly in the two treatment groups across the duration of the experiment. At the end of the experimental protocol, mean heart rate was 20 ± 7 bpm and 16 ± 3 bpm lower than at baseline in control and water-restricted SHR, respectively (Fig. 2b). In-depth analysis of this data by examination of hourly heart rate averages across the final week of the water restriction protocol revealed that heart rate was higher in water-restricted than control SHR in the hours before and during the 2-hour period of access to water. In contrast, during the remainder of the 24-hour period heart rate was similar in the two groups (Fig. 3b).

At baseline, locomotor activity was not significantly different between the control and water-restricted SHR. However, across the duration of the water restriction protocol we observed differences in locomotor activity between the treatment groups (Fig. 2c). This was associated with differences in activity patterns across a 24-hour period. Water-restricted rats were more active than their control counterparts in the hours preceding, and during, the period of access to water (Fig. 3c).

### 24-hour metabolic cage study

**Figure 4:**
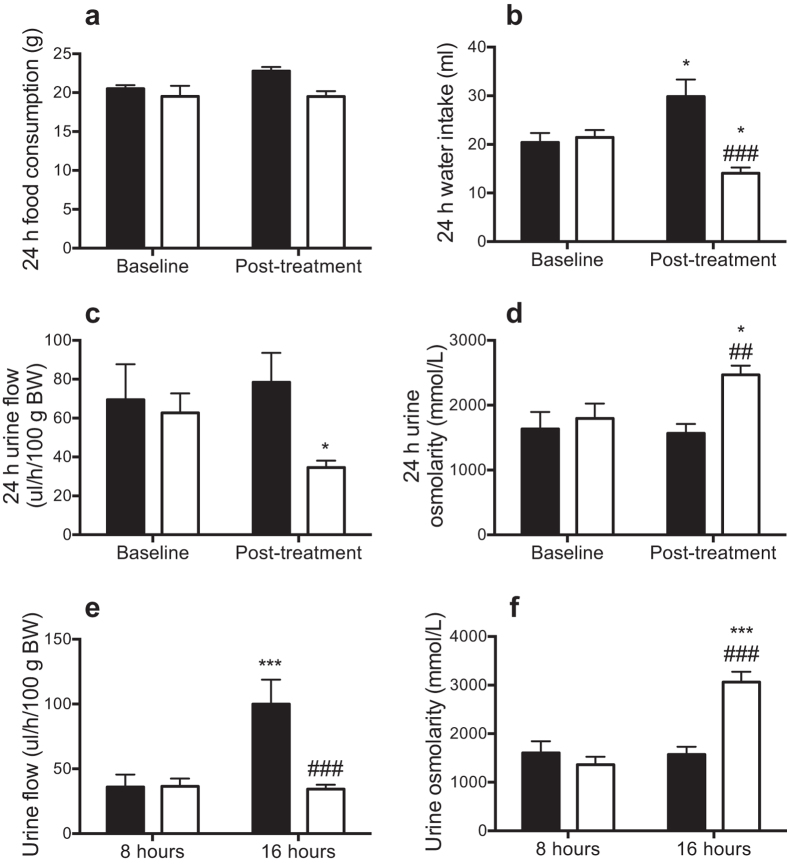
Food and water intake, urine flow and urine osmolarity data: (**a**) 24-hour food consumption, **(b)** water intake, **(c)** urine flow and **(d)** urine osmolarity in control (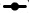) and water-restricted (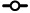) SHR at baseline and at the end of the 4-week water restriction protocol. (**e,f**) The 24-hour urine flow and osmolarity data for the post-treatment collection period are replotted as the first 8 hours and final 16 hours. Data are presented as mean ± SEM and were analyzed using repeated-measures ANOVA with Bonferroni’s post-hoc tests (2 comparisons). *P ≤ 0.05 and ***P ≤ 0.001 versus baseline or 8 hours. ^#^P ≤ 0.05, ^##^P ≤ 0.01 and ^###^P ≤ 0.001 versus control SHR. n = 8–13 per group.

Measurements were made from 9 am to 9 am at baseline and at the end of the 4 week protocol, with water access from 9–11 am in the water-restricted group. Food and water intake was similar in the control compared with the water-restricted SHR at baseline (Fig. 4a,b). At the end of the water restriction protocol, there was a trend for lesser food intake in water-restricted than control SHR (P = 0.08; Fig. 4a). Moreover, water intake was ~53% less in SHR subjected to water restriction than in control rats (Fig. 4b).

Urine flow was similar in control and water-restricted SHR at baseline (Fig. 4c). In the control SHR, urine flow was similar to baseline at the end of the 4-week treatment period. In contrast, as would be expected, 24-hour urine production was 34 ± 9% less than baseline levels in rats subjected to water restriction (Fig. 4c). Urine flow was also assessed as the first 8 hours (including 2-hour period of access to water) and the final 16 hours of urine collection. This analysis showed that urine production in the water-restricted SHR during the first 8 hours was similar to that observed in the control SHR. However, during the final 16-hour collection period (when water-restricted SHR had no access to water), urine flow was ~66% less in water-restricted than control SHR (Fig. 4e).

At baseline, 24-hour urine osmolarity was similar in the two treatment groups (Fig. 4d). In the control SHR, we did not observe any significant change in 24-hour urine osmolarity across the time course of the experiment. In contrast, 24-hour urine osmolarity was 69 ± 26% greater post-treatment than at baseline in water-restricted SHR (Fig. 4d). Moreover, urine osmolarity varied across the day in the water-restricted SHR. Urine osmolarity was similar in the control and water-restricted SHR during the first 8 hours of the urine collection. However, during the final 16 hours of the urine collection, urine osmolarity was ~95% greater in the water-restricted than control SHR (Fig. 4f). This demonstrates the occurrence of regular periods of dehydration and rehydration in the water-restricted SHR.

### Renal function and morphology

**Figure 5:**
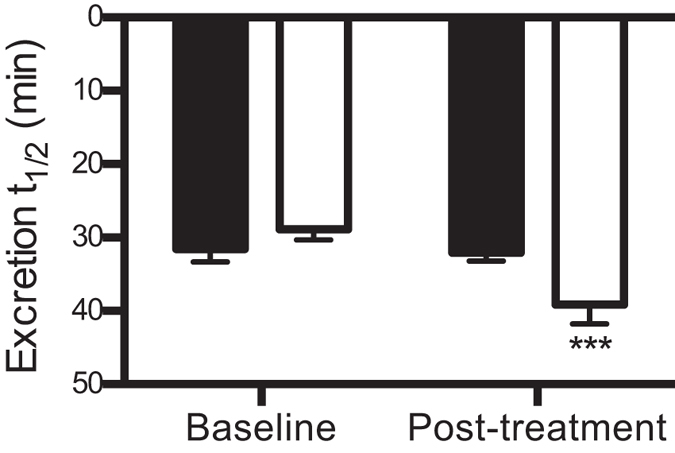
Assessment of glomerular filtration rate: Excretion half-life (t_1/2_) of FITC-sinistrin in control (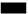) and water-restricted (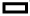) SHR at baseline and at the end of the 4-week water restriction protocol. Data are presented as mean ± SEM and were analyzed using repeated-measures ANOVA with Bonferroni’s post-hoc tests (2 comparisons). ***P ≤ 0.001 versus baseline. n = 8–10 per group.

Transcutaneous clearance of FITC-sinistrin, an estimate of GFR, was similar at baseline in the control and water-restricted SHR (Fig. 5). At the end of the 4-week water restriction protocol, no significant change in FITC-sinistrin clearance was observed in the control SHR compared to baseline. In comparison, FITC-sinistrin clearance measured at the end of the dehydration period prior to rehydration was prolonged by 39 ± 12% in the water-restricted SHR, which is representative of a significant decline in GFR (Fig. 5).

**Figure 6:**
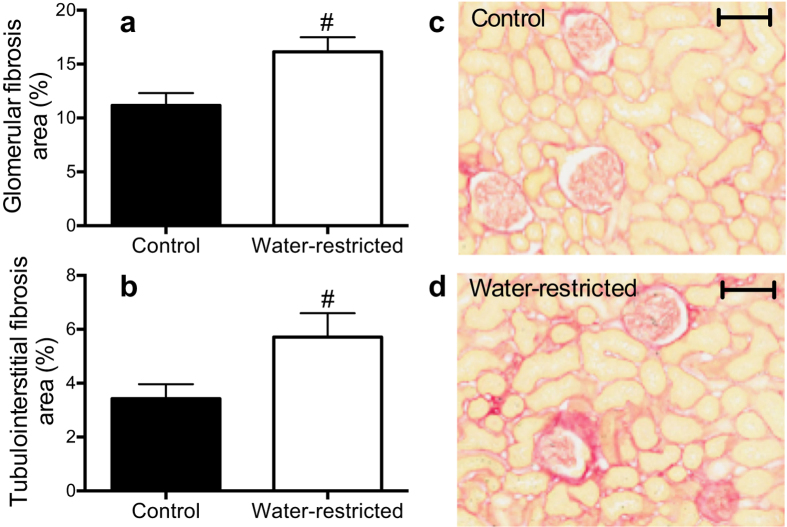
Assessment of renal fibrosis: Bar graphs represent quantification of the **(a)** glomerular and **(b)** tubulointerstitial percentage area positively stained with picrosirius red in control (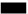) and water-restricted (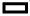) SHR. Representative images of renal cortical fibrosis in **(c)** control and **(d)** water-restricted SHR at the end of the 4-week water protocol. Scale represents 100 μm. Data are presented as mean ± SEM. Data were analyzed using an unpaired t-test. ^#^P ≤ 0.05 versus control SHR. n = 8–13 each.

Glomerular fibrosis, as assessed by picrosirius red staining, was ~45% greater in the water-restricted than control SHR (Fig. 6a). Similarly, we observed a ~68% greater proportion of tubulointerstitial fibrosis in kidney tissue from water-restricted SHR as compared to their control counterparts (Fig. 6b). Glomerular morphology, podocyte number and density, and expression levels of the podocyte-specific markers, p57 and synaptopodin, were not significantly different between the control and water-restricted SHR (see Supplementary Fig. S2).

The urinary concentration ratios of protein, uric acid and neutrophil gelatinase-associated lipocalin (NGAL) relative to creatinine were not significantly different between the groups at baseline (not shown). At the end of the 4-week treatment period, during the first 8 hours of urine collection when urine flow was not different between the groups, urinary protein/creatinine and uric acid/creatinine were similar between groups. However, urinary NGAL/creatinine was ~50% greater in the water-restricted compared to the control SHR (P < 0.05; see Supplementary Fig. S3). Urinary levels of NGAL, protein, and uric acid decreased significantly during the final 16 h of urine collection in the water restricted group, likely reflecting the marked reduction in urine flow (~70%) and GFR (~25%) during this time (Supplementary Fig. S3).

### Circulating and renal T cell populations and cytokine production

**Figure 7:**
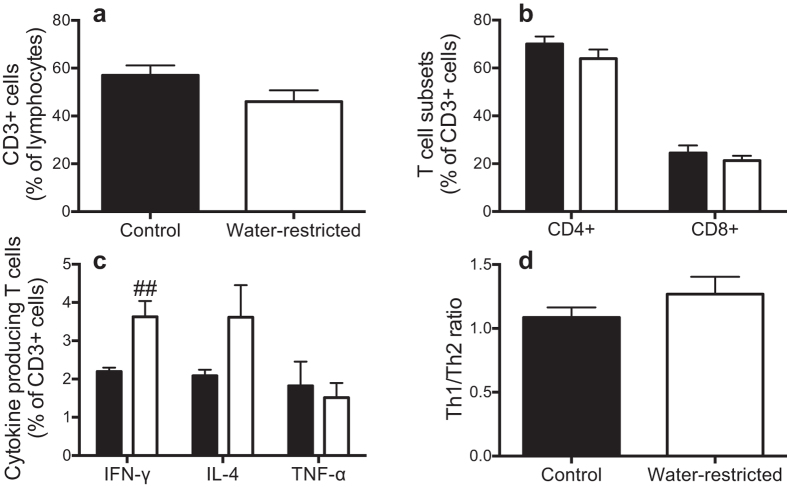
Circulating renal immune cell infiltration and cytokine production: Circulating renal immune cell infiltration and cytokine production in control (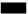) and water-restricted (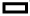) SHR at the end of the 4-week water restriction protocol. **(a)** Circulating T cells (CD3+ cells), the proportion of (**b)** circulating T helper cells and cytotoxic T cells and **(c)** T cells producing IFN-γ, IL-4 and TNF-α, and **(d)** Th1:Th2 proportion of cytokine producing T cells (measured as the ratio of IFN-γ/IL-4). Data are presented as mean ± SEM. Data were analyzed using an unpaired t-test. ^##^P ≤ 0.01 versus control SHR. n = 7–13 each.

Circulating CD3+ T cell count and the proportion of the T cell subpopulations characterized by CD4+ (T helper cells) and CD8+ (cytotoxic T cells) were similar in the control and water-restricted SHR (Fig. 7a,b). However, the expression profile of circulating T cell-derived cytokines differed between the treatment groups (Fig. 7c). There was ~1.6-fold more circulating IFN-γ-producing T cells in the water-restricted than control SHR. There also appeared to be more circulating IL-4-producing T cells in the water-restricted than control SHR although this apparent effect was not statistically significant (P = 0.1). Thus, there was no significant imbalance in the circulating Th1/Th2 ratio (measured as the ratio of IFN-γ/IL-4) between the control and water-restricted SHR (Fig. 7d). Furthermore, the proportion of circulating tumour necrosis factor-α (TNF-α) producing T cells was similar between the treatment groups (Fig. 7c).

**Figure 8:**
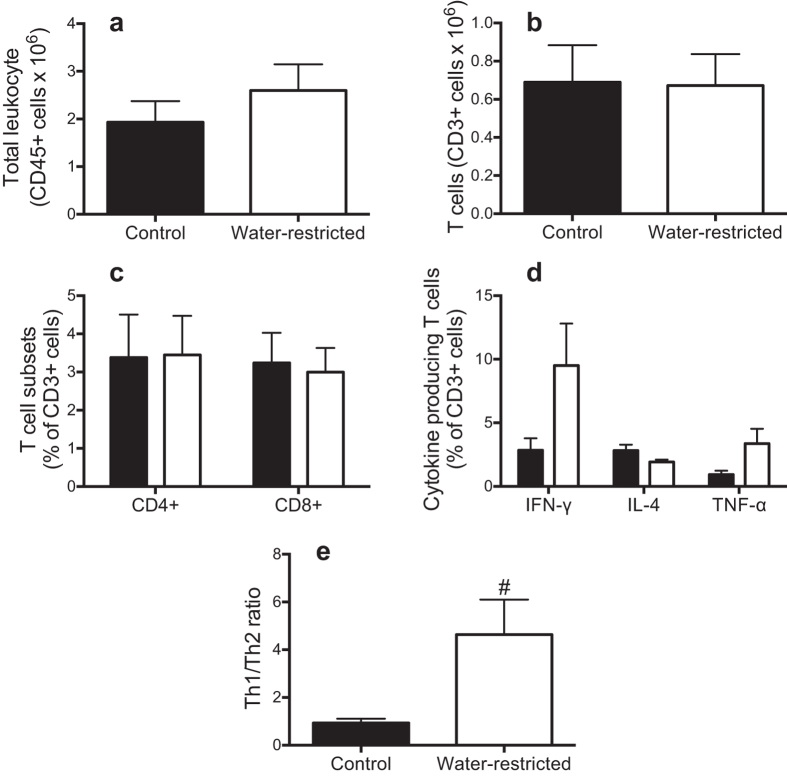
Renal immune cell infiltration and cytokine production: Renal immune cell infiltration and cytokine production in control (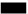) and water-restricted (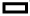) rats at the end of the 4-week water restriction protocol. Renal **(a)** leukocyte (CD45+ cells) **(b)** T cells (CD3+ cells), the proportion of **(c)** renal T helper cells and cytotoxic T cells and **(d)** T cells producing IFN-γ, IL-4, and TNF-α **(e)** the Th1:Th2 proportion of cytokine producing T cells (measured as the ratio of IFN-γ/IL-4). Data are presented as mean ± SEM. Data were analyzed using an unpaired t-test. ^#^P ≤ 0.05 versus control SHR. n = 10–17 each.

In the kidney, leukocyte and T cell infiltration was similar between the control and water-restricted SHR (Fig. 8a,b). Moreover, there was no significant difference in the proportion of the T cell subpopulations characterized by CD4+ (T helper cells) and CD8+ (cytotoxic T cells) between the treatment groups (Fig. 8c). However, there was a phenotypic shift towards a pro-inflammatory Th1 (IFN-γ-producing) phenotype in kidney T cell infiltrate from the water-restricted SHR. There was no statistically significant difference in the proportion of renal T cells producing IFN-γ or IL-4 between the water-restricted and control SHR (P = 0.07 and P = 0.09, respectively) (Fig. 8d). However, the renal Th1/Th2 ratio (measured as the ratio of IFN-γ/IL-4) was ~5-fold greater in SHR subjected to water-restriction than control SHR (Fig. 8e). There was also a trend for a greater proportion of renal TNF-α-producing T cells in water-restricted as compared to control SHR (P = 0.07) (Fig. 8d).

## Discussion

The key findings of this study were that daily cycles of dehydration and replenishment induced by periodic water restriction exacerbated hypertension, decreased renal function, and increased NGAL excretion, renal inflammation and fibrosis in male SHR. These data provide strong evidence that recurrent dehydration associated with chronic periodic water intake hastens the progression of CKD and aggravates hypertension.

Detailed investigations into the impact of recurrent dehydration associated with periodic water intake are long overdue. It is very common for individuals to go through daily cycles of mild dehydration and replenishment due to the time-consuming nature and physical demands of their work and home schedules, or simply out of habit. Alternatively, for other individuals irregular water intake is a means of dealing with incontinence, a result of incapacity to acquire or consume fluids, or a clinically recommended practice to avoid fluid overload and hyponatremia during the latter stages of CKD. Importantly, our water restriction protocol in rats allows us to interrogate the impact of recurrent dehydration on kidney health. Under conditions of dehydration, there is a marked increase in endogenous circulating levels of the hormone arginine vasopressin (AVP)^18^. As demonstrated in the current study, restriction of water intake to a 2-hour window each day induced periods of high and low urine osmolarity. It seems reasonable to propose that these fluctuations were associated with fluctuations in the plasma concentration of AVP since urine osmolarity is highly correlated with plasma AVP^19^. Such variation in urine osmolarity is commensurate with the physiological results of cyclic changes in daily water intake in humans^20^.

Our data provide strong evidence that recurrent dehydration associated with chronic periodic water intake promotes hypertension and the progression of CKD. Our key findings were that periodic water intake exacerbated hypertension and promoted renal dysfunction and injury in male SHR. Specifically, arterial pressure gradually increased in SHR subjected to periodic water restriction, an effect that was observed approximately 2 weeks into the 4-week water restriction protocol. Other studies have also suggested an association between hydration status and hypertension^9^,^21^. In addition, in the final week of water restriction a trend for a smaller difference in day-night arterial pressure was observed in the water-restricted SHR. This was likely due to differences in arterial pressure at specific time points across a 24-hour period between the treatment groups. Indeed, greater spikes in arterial pressure were observed in the water-restricted than control group immediately prior to and during the water access period. However, it is also possible that this difference reflects activation of hormonal pathways (e.g. AVP; renin-angiotensin system, RAS) under conditions of water deprivation. Indeed, activation of the RAS has been shown previously to invert the circadian pattern of arterial pressure^22^.

Water restriction also led to an ~25% reduction in GFR, as indicated by an increase in FITC-sinistrin elimination half-life. This measurement was made at the end of the dehydration period prior to rehydration. Thus, the reduction in GFR could reflect CKD or an acute event as a result of the dehydration. In future studies it will be important to determine GFR following rehydration. If GFR were normalized following rehydration this would suggest that repetitive bouts of renal ischemia inducing acute kidney injury might play a role in the development of CKD. It would also be of interest in future studies to track changes in GFR using the transcutaneous assessment of FITC-sinistrin clearance across the duration of the study in order to identify the temporal progression of recurrent-dehydration induced alterations in kidney function. In combination with radiotelemetric assessment of arterial pressure, this would provide us with significant insight into the time-course of pathological changes induced by recurrent dehydration associated with periodic water intake, and whether the reduction in GFR is secondary to the increase in arterial pressure. Cowley and colleagues recently demonstrated the strength of combining such methods to follow the progression of disease^23^.

Notably, the exacerbation of hypertension and decline in kidney function in water-restricted SHR was associated with glomerular and tubulointerstitial fibrosis. This was not associated with increased urinary protein excretion at least after 4-weeks of chronic water restriction. Nor was there any evidence of damage to the glomerular filtration barrier, as documented by no change in podocyte number and density, synaptopodin and P57 expression in the water-restricted SHR. However, NGAL excretion was increased in association with the greater tubulo-interstitial fibrosis in the water-restricted SHR, similar to the findings of Roncal Jimenez *et al*.^24^, in a heat-stress induced model of dehydration in mice. Together this evidence suggests that recurrent dehydration induces tubulo-interstitial injury and fibrosis. Furthermore, clear evidence now exists that inflammation and immune system activation play an active role in the development and progression of CKD^25^ and, in the context of the present study, has previously been shown to precede the pathogenesis of renal damage in SHR^14^. In this regard, we also observed a phenotypic change in the T cells infiltrating the kidney in water-restricted SHR, resulting in a greater renal Th1/Th2 ratio than in SHR with free access to water, which may drive macrophage recruitment. Indeed, previous studies have indicated that macrophage infiltration may play a role in dehydration related CKD^24^. Thus, our data suggest that T-cell activation of the immune system may recruit inflammatory cells to the kidney contributing to the promotion of renal dysfunction and damage in SHR subjected to water restriction. Future studies to determine the mechanistic pathways linking T-cell activation, renal injury and hypertension in response to recurrent dehydration are now required.

It is also of relevance that water-restricted SHR gained less weight than control SHR across time in the current study. This may be attributable to differences in food intake between the water-restricted and control SHR. Both of our treatment groups were given ad libitum access to food. We observed a trend for less food intake in water-restricted than control SHR. Although we did not monitor timing of food intake, previous investigations have found that rats subjected to water restriction first drink when access to water is restored, and then alternate drinking and eating^26^. Therefore, the majority of food intake in water-restricted SHR likely occurred during the period of water access. However, the difference in weight gain between the treatment groups across the duration of the study may also be attributable to differences in energy expenditure, basal metabolic rate and/or a consequence of reduced total body water content associated with reduced 24-hour water intake in water-restricted SHR. In consequence, based on our study findings to date, we cannot rule out the possibility that the lower food consumption and thus energy intake in the water-restricted than control SHR induced adverse metabolic consequences. These in turn may impair optimal function of the immune system and contribute to the aggravation of hypertension and renal injury that we observed in the water-restricted SHR; independent of the effects of dehydration. Accordingly, such differences in nutritional status require careful consideration in future investigations.

Hyperuricemia, elevated serum levels of uric acid, has also been proposed as a potential mechanism whereby recurrent dehydration might lead to CKD^27^. Hyperuricemia is a common observation in individuals with CKD^28^,^29^,^30^. Furthermore, it is also considered a potential causative factor in the development and progression of CKD via a range of pathogenic mechanisms including, but not limited to, activation of the RAS, and promotion of endothelial dysfunction, inflammation and oxidative stress^28^,^29^. A large proportion of cases of hyperuricemia are attributable to impaired renal excretion of uric acid. However, increased production of uric acid, or a combination of increased production and altered urinary excretion of uric acid can also contribute^28^. Based on our observation in the present study that urinary uric acid levels were not different between the control and water-restricted SHR (as measured when the animals were water replete and urine flow was similar between the groups), it is plausible that altered renal handling of uric acid could possibly be a contributing factor to the exacerbation of hypertension and promotion of renal dysfunction and damage in water-restricted SHR. However, as recently proposed by Johnson *et al*.^29^, since the kidney is the major organ responsible for the excretion of uric acid, a reduction in GFR contributes to an elevation in serum levels of uric acid. Moreover, it is also well recognized that dietary factors can affect serum uric acid levels^28^. Therefore, given that we observed a trend for less food intake in water-restricted than control SHR, this might also have influenced uric acid excretion between the treatment groups. Accordingly, future studies in which serum uric acid levels are monitored and uric acid levels are modulated would be essential to uncover a causal role for uric acid in the setting of recurrent dehydration-induced CKD and interrogate the underlying mechanistic pathways that contribute. Moreover, it is important to consider in this and future investigations that uric acid metabolism differs significantly between humans and other species, including rodents. In humans, uric acid is the end product of purine metabolism. Conversely, uric acid is further metabolized to allantoin in rats by the enzyme, uricase. As a result, rats have lower serum levels of uric acid than humans. Thus, information gained from studies of uric acid metabolism in rats should be extrapolated to humans with caution.

Our data add to evidence that hydration status has a major impact on the progression of renal dysfunction and disease. Several studies have reported a protective effect of reducing urine concentrating activity by increasing water intake, or administering AVP receptor antagonists, on the progression of CKD in rats^10^,^31^,^32^,^33^. Moreover, AVP infusion accelerated the progression of CKD in 5/6 nephrectomized AVP-deficient Brattleboro rats with diabetes insipidus^34^.

In addition, the knowledge generated from our study extends the previous investigations of Roncal Jiminez *et al*. into the impact of recurrent heat-induced dehydration on cardiovascular and renal function in normotensive mice^24^. Mice that were subjected to heat stress in combination with restricted water access (enforcing delayed rehydration) developed renal dysfunction and injury, and demonstrated greater arterial pressure than control mice subjected to heat stress in the absence of delayed rehydration. For the first time, we have shown that recurrent dehydration associated with periodic water intake, in the absence of heat exposure, is sufficient to cause a significant rise in arterial pressure and renal dysfunction and morphological changes, at least in male SHR.

There is also some evidence that sustained increases in urine osmolarity are associated with alterations in renal function and morphology in humans, suggesting that recurrent dehydration associated with periodic water intake promotes CKD in the clinical situation. Meijer *et al*. identified an association between the concentration of plasma copeptin, a marker of endogenous AVP release, and urinary albumin excretion and microalbuminuria in the general population^35^ and accelerated decline in renal function in renal transplant recipients^36^. In addition, epidemiological evidence suggests that low water intake correlates with an increased risk of CKD. Clark *et al*. examined the relationship between urine volume and renal decline over 6 years in a large Canadian cohort and identified a faster decline in estimated GFR in individuals with low as compared to high urine volume^1^. Similarly, Strippoli *et al*. reported an inverse relationship between intake of fluid and prevalence of CKD based on the findings of two cross-sectional surveys in Australia^3^. Moreover, Sontrop *et al*. analyzed data from the National Health and Nutrition Examination Survey (NHANES) from a representative sample of the US population and found that the prevalence of stage III CKD was highest amongst individuals with the lowest water intake^2^. In light of these observations, a clinical trial is presently underway to ascertain the impact of increased water intake on renal decline in patients with CKD.

Notably, evidence also exists that low urine flow may promote hypertension in humans, or at least worsen pre-existing hypertension, by reducing urinary sodium excretion. Bankir *et al*. demonstrated an association between impaired urine flow and higher arterial pressure in patients with diabetes^9^. Moreover, Choukroun *et al*. showed that low water intake limits the capacity of the kidneys to excrete a moderate sodium load (less than 50% of their usual daily intake) in male healthy volunteers^21^.

To further advance this field, future studies should further interrogate the underlying mechanisms that are activated by recurrent dehydration associated with periodic water intake, and how they drive the development of CKD. This could arm us with the knowledge of downstream mechanisms that could be targeted to prevent or arrest the clinical course of CKD in individuals who habitually or intentionally demonstrate irregular water consumption. In this regard, it is plausible that key hormones, including AVP and the major RAS effector peptide, angiotensin II (AngII), produced during dehydration, play a significant role. Such hormones elicit significant effects on arterial pressure, glomerular hemodynamics and non-hemodynamic renal mechanisms^37^,^38^; all of which might induce alterations in renal function and morphology in the setting of chronic recurrent dehydration. The recent investigation by Roncal Jiminez and colleagues provided evidence that activation of the aldose reductase pathway in the renal cortex could also contribute to the development of recurrent dehydration-induced CKD via the generation of endogenous fructose in the kidney, which might cause renal injury via metabolism by fructokinase^24^. Accordingly, it would be insightful in future studies to assess time-dependent changes in AVP and AngII levels, and renal sorbitol and fructose levels, to look for correlations between peak changes in and/or chronic activation of these pathways with the promotion of hypertension (including any impact on circadian rhythm of arterial pressure) and aggravation of renal injury and dysfunction under conditions of recurrent dehydration. It will be of major interest in future studies to include female cohorts, as well as populations without existing disease (e.g. normotensive animals) and advanced stages of CKD. This would likely provide insight into whether the impact of recurrent dehydration on the development and progression of renal dysfunction and injury is influenced by sex and/or the prior existence of renal disease. Moreover, investigations in normotensive populations could also assist in the dissection of the underlying mechanisms driving renal injury.

In conclusion, our findings support the notion that chronic recurrent dehydration associated with irregular water intake is a risk factor for the progression of CKD. This highlights the importance of regular daily water intake for the maintenance of kidney health in populations with existing cardiovascular and renal disease, and provides further impetus for studies of this phenomenon in human populations at risk of CKD. Importantly, our findings are consistent with the proposition that repetitive bouts of acute kidney injury, induced by recurrent dehydration, may activate the immune system and promote progression to CKD. Further mechanistic studies are now required to provide insight into the underlying pathways that drive the progression of recurrent dehydration-induced kidney disease and thus uncover potential therapeutic targets. Moreover, it will also be of major interest to investigate this phenomenon in females as well as males, and populations without existing disease.

## Methods

### Animals

Ten-week old male SHR were obtained from the Animal Resources Centre (Canning Vale, Western Australia, Australia). Rats were housed individually under standard laboratory conditions (12-hour light/dark cycle at a temperature of 21 °C) and were fed a sodium-controlled diet (0.25% w/w sodium chloride; Specialty Feeds, Glen Forrest, WA, Australia) ad libitum. Experiments were approved by the Monash University, School of Biomedical Sciences Animal Ethics Committee and were performed in accordance with the Australian Code of Practice for the Care and Use of Animals for Scientific Purposes. Rats were allowed 1–2 weeks to acclimatize to these conditions prior to the commencement of the study protocol.

### Baseline renal function and mean arterial pressure

At 11–12 weeks of age, rats were placed in individual metabolic cages for 24-hours to collect a 24-hour urine sample for assessment of baseline renal excretory function and hydration status (urine osmolarity). Specifically, food and water consumption and urine output were recorded, and urine samples were collected for subsequent analyses of urine osmolarity (Advanced Osmometer 2020, Advanced Instruments, Needham Heights, MA, USA).

The following day, baseline glomerular filtration rate (GFR) was determined via the transcutaneous clearance of fluorescein isothiocyanate (FITC)-labeled sinistrin using a miniaturized non-invasive clearance (NIC)-kidney fluorescent detection device (Mannheim Pharma & Diagnostics GmbH, Mannheim, Germany)^39^. Briefly, rats were lightly anesthetized (2–2.5% v/v isoflurane). The NIC-Kidney device was attached to a depilated region on the back of the rat using a double-sided adhesive patch and adhesive tape. Following a 3–5 minute baseline period, a bolus of FITC-sinistrin (3 mg/100 g made up in 0.9% sodium chloride solution) was administered via tail the vein. The rat was then returned to an experimental chamber for 2 hours. At the end of the 2-hour recording period, rats were lightly anesthetized and the NIC-kidney device was removed. The collected data were subsequently analyzed using NIC-kidney device partner software. The software generates the elimination kinetics curve of FITC-sinistrin from which excretion half-life (t_1/2_) determinations, which correlate with GFR, were calculated using a one-compartment model.

Upon completion of baseline metabolic cage studies and GFR measurements, each rat was anesthetized (isoflurane; 2–5% v/v O_2_) and a radiotelemetry probe (PA-C40, Data Sciences International, MN, USA) was implanted into the abdominal aorta for the measurement of mean arterial pressure (MAP), as previously described^22^. After a 10-day recovery period, baseline MAP was recorded continuously for 3 days.

### Four-week water treatment protocol

Rats were randomly allocated to 1 of 2 treatment groups (control or water-restricted) and the 4-week treatment protocol commenced. Across the 4-week treatment period, control rats were given unlimited access to water. In contrast, water-restricted rats were given access to water for only a 2-hour period (9 am to 11 am) each day. Water intake for all rats was recorded daily. MAP was measured continuously across the entire 4-week treatment period.

At the end of the water treatment protocol, urinary metabolic cage studies and GFR measurements were repeated. GFR measurements were taken in water-restricted rats prior to their daily period of access to water. During the final 24-hour urinary metabolic cage study, urine was collected at the 8-hour and 24-hour time points to enable us to examine differences in urine production and concentration across a 24-hour time period, and also during the periods when the rats did and did not have access to water. Furthermore, urinary creatinine (Exocell, Excel Inc, PA, USA), protein (Bio-Rad Protein assay, Bio-Rad Laboratories Pty. Ltd., NSW, Australia), uric acid (Beckman Coulter SYNCHRON DXC800, Beckman Coulter, NSW, Australia) and NGAL (R&D systems Rat Lipocalin-2/NGAL DuoSet DY3508) concentrations were determined. The concentrations of these biomarkers were expressed relative to creatinine concentration.

### Tissue harvesting and preparation for flow cytometry

At the end of the study, rats were humanely killed via carbon dioxide asphyxiation and a 1 ml blood sample was collected via cardiac puncture. Rats were then intracardially perfused with phosphate buffered saline (PBS; NaCl 137 mmol/L, KCl, 2.7 mmol/L, Na_2_HPO_4_ 10 mmol/L, KH_2_PO_4_ 2 mmol/L), and the kidneys were removed. Blood samples were mixed with red blood cell (RBC) lysis buffer (NH_4_Cl 155 mmol/L, KHCO_3_ 10 mmol/L, EDTA 0.01 mmol/L) to remove all erythrocytes, and were then washed in PBS and cells were counted using a Countess® Automated Cell Counter (Life Technologies).

To isolate kidney mononuclear cells, the left kidney was enzymatically digested using collagenase type IX (125 U/mL), hyaluronidase (60 U/mL) and collagenase type I-S (450 U/mL; all enzymes from Sigma Aldrich) dissolved in PBS buffer containing calcium and magnesium for 45 min at 37 °C as previously described^40^. Samples were then passed through a 70 μm cell strainer (BD Biosciences) to yield a single cell suspension. After washing with PBS, cells were centrifuged at 1200 RPM for 10 min at 4 °C. Samples were then resuspended in 40% w/v isotonic percoll solution (GE Healthcare), and 60% w/v isotonic percoll was gently underlaid beneath the sample for density centrifugation. Density gradients were then spun at 2700 RPM for 20 min at room temperature with the brake off. Mononuclear cells were isolated from the interface of the percoll layers, and washed with PBS. Blood and kidney mononuclear cells were stained with an aqua live/dead viability stain (Life Technologies) for 15 minutes at 4 °C. After washing with FACS buffer (PBS with 0.5% bovine serum albumin) cells were stained with fluorochrome-conjugated antibodies (all from Biolegend) for surface markers including CD45 (leukocytes; clone OX-1; PE-Cy7), CD3 (T cells; clone 1F4; BV605), CD4 (T-helper cells; clone W3/25; APC-Cy7) and CD8 (cytotoxic T cells; clone OX-8; PerCP). Cells were analyzed using an LSR II flow cytometer (BD Biosciences) to quantitatively characterize T cell populations and tissue-infiltrating T cells, as previously described^40^.

### Intracellular cytokine analyses

Blood and kidney samples were prepared as described above. Following preparation of mononuclear cells and digestions, cells were resuspended in complete RPMI1640 media (fetal bovine serum, 10%; streptomycin/penicillin, 100 U/mL; HEPES, 25 mmol/L; 2-mercaptoethanol, 2 μM) and seeded onto a 96-well plate. Cells were then stimulated with phorbol-12-myristate (PMA; 50 ng/mL) and ionomycin (500 ng/mL) in the presence of Golgi transport inhibitors brefeldin A and monensin (BD Biosciences) for 6 hours at 37 °C with 95% O_2_ and 5% CO_2_. Following stimulation, cells were washed and stained with a viability stain and surface markers as described above. Cells were also fixed and permeabilized, and stained for intracellular cytokines interferon-γ (IFN-γ; clone DB-1; FITC; Biolegend), tumour necrosis factor-α (TNF-α; clone TN3-19.12; PE; Biolegend), and interleukin (IL)-4 (clone OX-81; eFluor660; Affymetrix). Cells were analyzed using a LSR II flow cytometer (BD Biosciences). Representative flow cytometric plots of the gating strategy employed for circulating and renal cytokine-producing T cells are shown in Supplementary Fig. S4. Gating for all cytokines was defined by an unstimulated blood or renal sample run concurrently with each assay (see Supplementary Fig. S5). FlowJo Software Version 10.0.6 (Tree Star) was used to analyze all flow cytometric data.

### Renal fibrosis

For assessment of collagen deposition, the right kidney was fixed in 10% neutral buffered formalin and paraffin embedded. Renal glomerular and tubulointerstitial fibrosis was then analyzed in 4 μm-thick paraffin-embedded kidney sections stained with 0.05% w/v Picrosirius red solution. Specifically, digital images were captured at 40x magnification using an Aperio Scanscope AT Turbo scanner in a blinded manner. Forty random glomeruli and 40 non-overlapping cortical fields without vessels or glomeruli were then analyzed for each kidney and the fraction of the stained (red) areas was determined using Aperio Image Scope software. Results are presented as percentage of glomerular or tubulointerstitial area stained with Picrosirius red.

### Immunofluorescence

Antigen retrieval was performed on 4 μm thick sections using Dako Target Retrieval Solution (S1699; Dako, Glostrup, Denmark) on a Dako PT Link Instrument (PT10126; Dako) for 30 minutes at 98 °C. Sections were then allowed to cool. A standard immunofluorescence protocol was performed as previously reported^41^. Briefly, sections were incubated in primary and secondary antibodies for 1 hour at room temperature with five washes with DakoWash Buffer (K8007; Dako) between each incubation step. In order to assess glomerular and podocyte morphology we used a polyclonal rabbit anti–mouse p57 (1:200; SC8298; Santa Cruz Biotechnology, Santa Cruz, CA) and a goat anti–mouse SNP (1:400; SC21537; Santa Cruz Biotechnology) as primary antibodies and a polyclonal donkey anti–rabbit Alexa Fluor 555 (1:200; A31572; Life Technologies) and a polyclonal chicken anti–goat Alexa Fluor 488 (1:400; A2467; Life Technologies) as secondary antibodies. After incubation with secondary antibodies, sections were washed five more times with DakoWash Buffer, and DAPI (1:10,000; D1306; Life Technologies, Carlsbad, CA) was added for 20 minutes. Five final washes were done before coverslipping each section with Prolong Gold (P36934; Invitrogen, Carlsbad, CA).

### Confocal microscopy and model-based stereology

Optical images from 4 μm sections were obtained using an inverted Leica SP5 Laser Confocal Microscope (Leica Microsystems) fitted with a 403 objective lens (1.25 numerical aperture) with a 1X zoom using sequential imaging for 405, 488, and 555 nm. Representative images were obtained with six line averages and stored in a 1024 × 1024 pixel frame. Glomerular cross-sections were sampled using a systematic walk from the superficial to the inner cortex. These images were used to estimate glomerular volume and total podocyte number per glomerulus by the Weibel and Gomez method^42^. Podocyte density was then calculated by dividing total podocyte number per glomerulus and glomerular volume.

### Statistical analyses

Data are presented as mean ± standard error of the mean (SEM). Body weight, daily water consumption, GFR and telemetry and metabolic cage study data were analyzed using repeated-measures analysis of variance (ANOVA) using the factors group (control or water-restricted SHR), time and their interaction. Bonferroni’s post-hoc tests were performed when multiple comparisons were made. Student unpaired t-tests (with Welch’s correction) were used for group comparisons of body weight, kidney weight, arterial pressure, heart rate, locomotor activity, renal fibrosis, and urinary uric acid excretion at baseline and/or at the completion of the experimental protocol. Two-tailed *P* values ≤ 0.05 were considered statistically significant.

## Additional Information

**How to cite this article**: Hilliard, L. M. *et al*. Chronic recurrent dehydration associated with periodic water intake exacerbates hypertension and promotes renal damage in male spontaneously hypertensive rats. *Sci. Rep.* **6**, 33855; doi: 10.1038/srep33855 (2016).

## Supplementary Material

Supplementary Information
