# Supplementary material for: Chronic recurrent dehydration associated with periodic water intake exacerbates hypertension and promotes renal damage in male spontaneously hypertensive rats
Source: Sci Rep. 2016 Sep 22;6:33855. doi: 10.1038/srep33855 (PMC5032121; doi:10.1038/srep33855)
Supplement: Supplementary Information [file srep33855-s1.pdf]

CHRONIC RECURRENT DEHYDRATION ASSOCIATED WITH PERIODIC WATER INTAKE EXACERBATES HYPERTENSION AND PROMOTES RENAL DAMAGE IN MALE SPONTANEOUSLY HYPERTENSIVE RATS

Lucinda M Hilliard[1], Katrina M Mirabito Colafella[1], Louise L Bulmer[1], Victor G Puelles[2], Reetu R Singh[1], Connie PC Ow[1], Tracey Gaspari[3], Grant R Drummond[3], Roger G Evans[1], Antony Vinh[3#], Kate M Denton[1#]*.

Cardiovascular Disease Program, Biomedicine Discovery Institute and Department of Physiology[1], Department of Anatomy and Developmental Biology, and Department of Pharmacology[2], Monash University, Melbourne, Victoria, Australia 3800. #These authors contributed equally to this work. *Indicates corresponding author.

Running title: Recurrent dehydration promotes renal damage in male SHR

Corresponding author:

Prof Kate Denton

Department of Physiology

26 Innovation Walk (Building 13F), Monash University

Victoria, Australia, 3800

Email: kate.denton@monash.edu

Phone: 61 3 9905 9553   Fax: 61 3 9905 2547

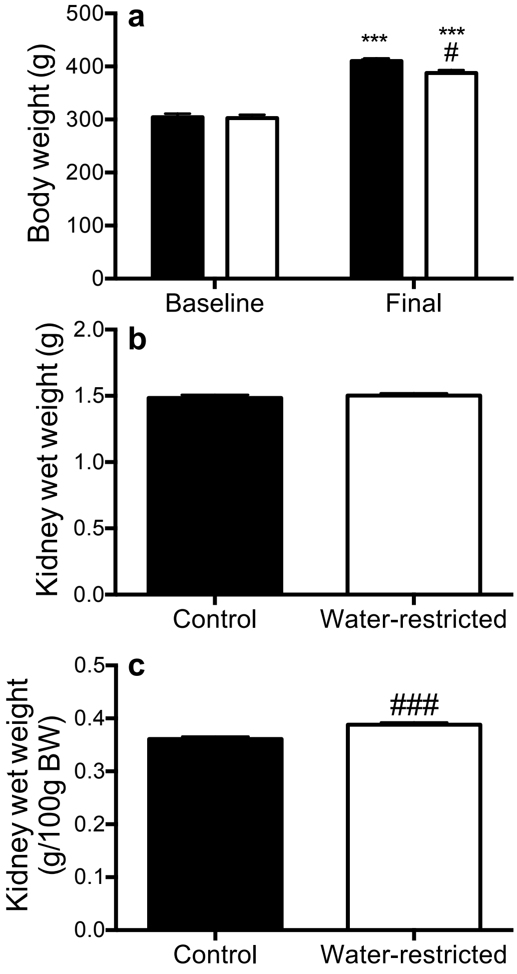

Supplementary Figure S1. Baseline characteristics: (a) Body weight in control (■) and water-restricted (□) SHR at baseline and at the end of the 4-week water-restriction protocol (post-treatment). **(b and c)** Kidney weight expressed in g and g per 100 g body weight in control and water-restricted SHR at the conclusion of the 4-week water restriction protocol. All data are presented as mean ± SEM. Body weight data were analyzed using repeated-measures ANOVA followed by Bonferroni's post hoc tests (2 comparisons per analysis). Kidney weight data were analyzed using an unpaired t-test. *** $P<0.001$ versus baseline. # $P<0.05$ versus control SHR. n = 8-13 per group.

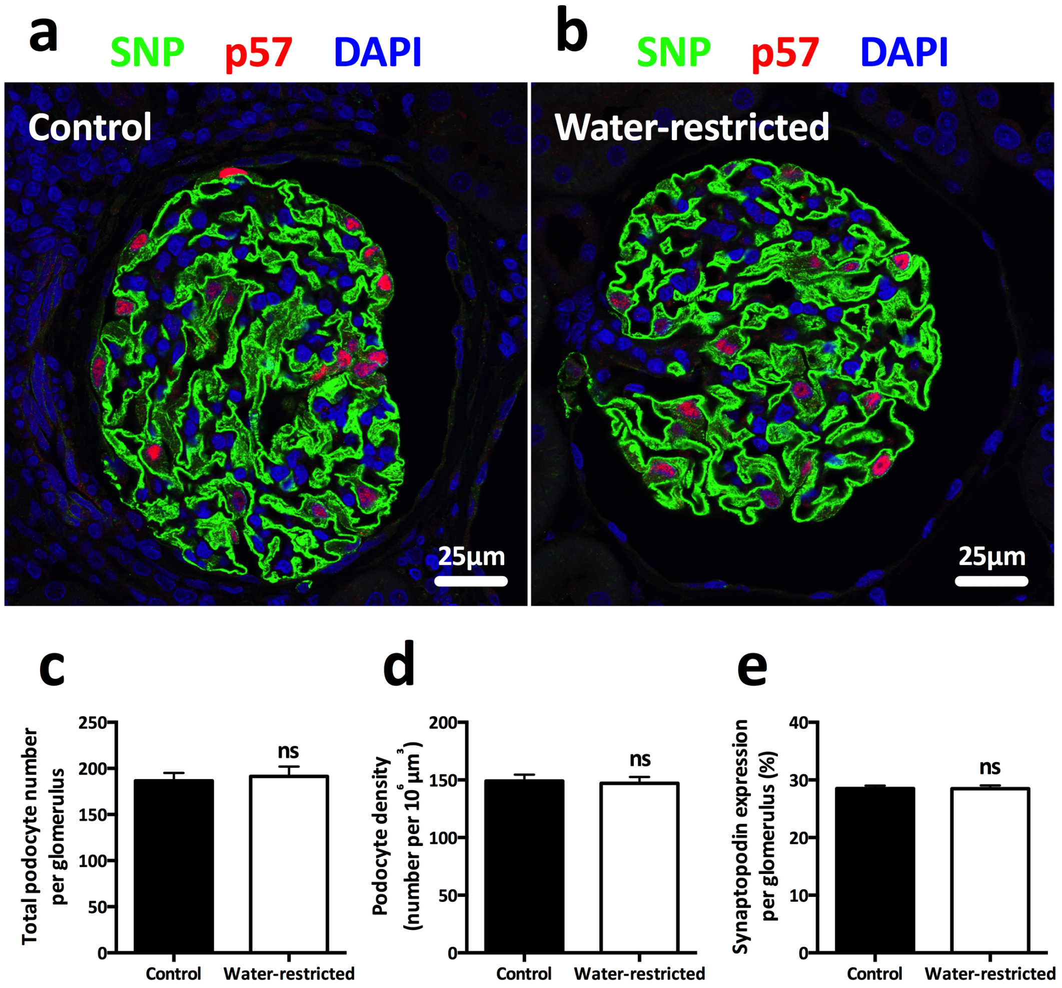

Supplementary Figure S2. Representative confocal images from (a) control (▬), and (b) water-restricted (▭) rats show normal glomerular morphology and comparable expression levels of podocyte-specific markers such as p57 (red) and synaptopodin (SNP, green). Total podocyte number per glomerulus (c), podocyte density (d) and synaptopodin expression (e) were similar between control (n=3) and water-restricted (n=3) rats. All data are presented as mean ± SEM. In each rat, 20 glomeruli were systematically sampled across the renal cortex (from superficial to juxtamedullary regions) for a total of 60 glomeruli analysed per group. Data were analysed using an unpaired t-test; ns: not significant.

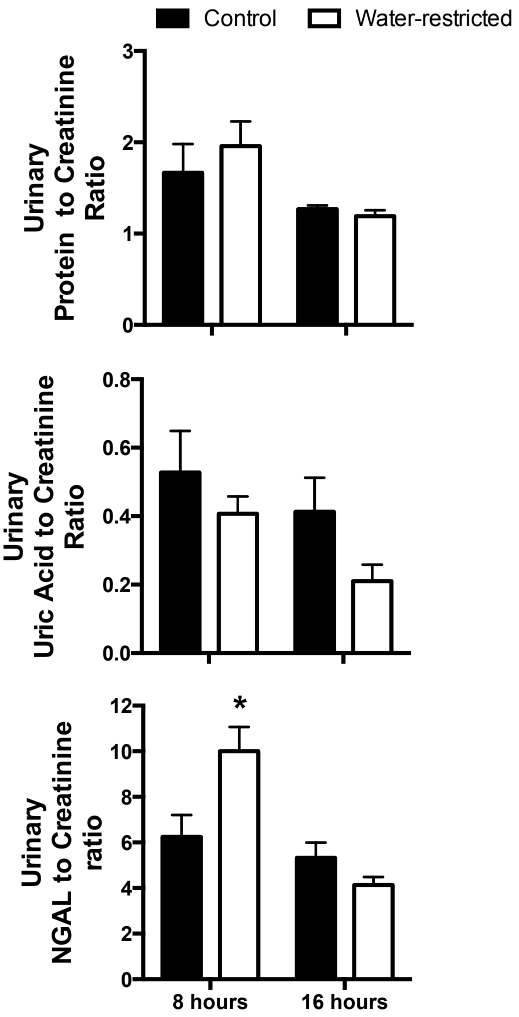

Supplementary Figure S3. Urinary protein, uric acid and NGAL concentration ratios: Urinary **(a)** protein, **(b)** uric acid and (**c**) NGAL concentrations are expressed as a ratio of creatinine concentration in control (▬) and water-restricted (▭) SHR at the end of the 4-week water-restriction protocol. Measurements were made during the first 8h (including the 2 hour period of water access) and last 16h of a 24h urine collection. Data are presented as mean ± SEM and were analyzed using repeated-measures ANOVA with Bonferroni's post-hoc tests (2 comparisons). * P≤0.0.5 versus control SHR at that time period. n = 8-13 per group.

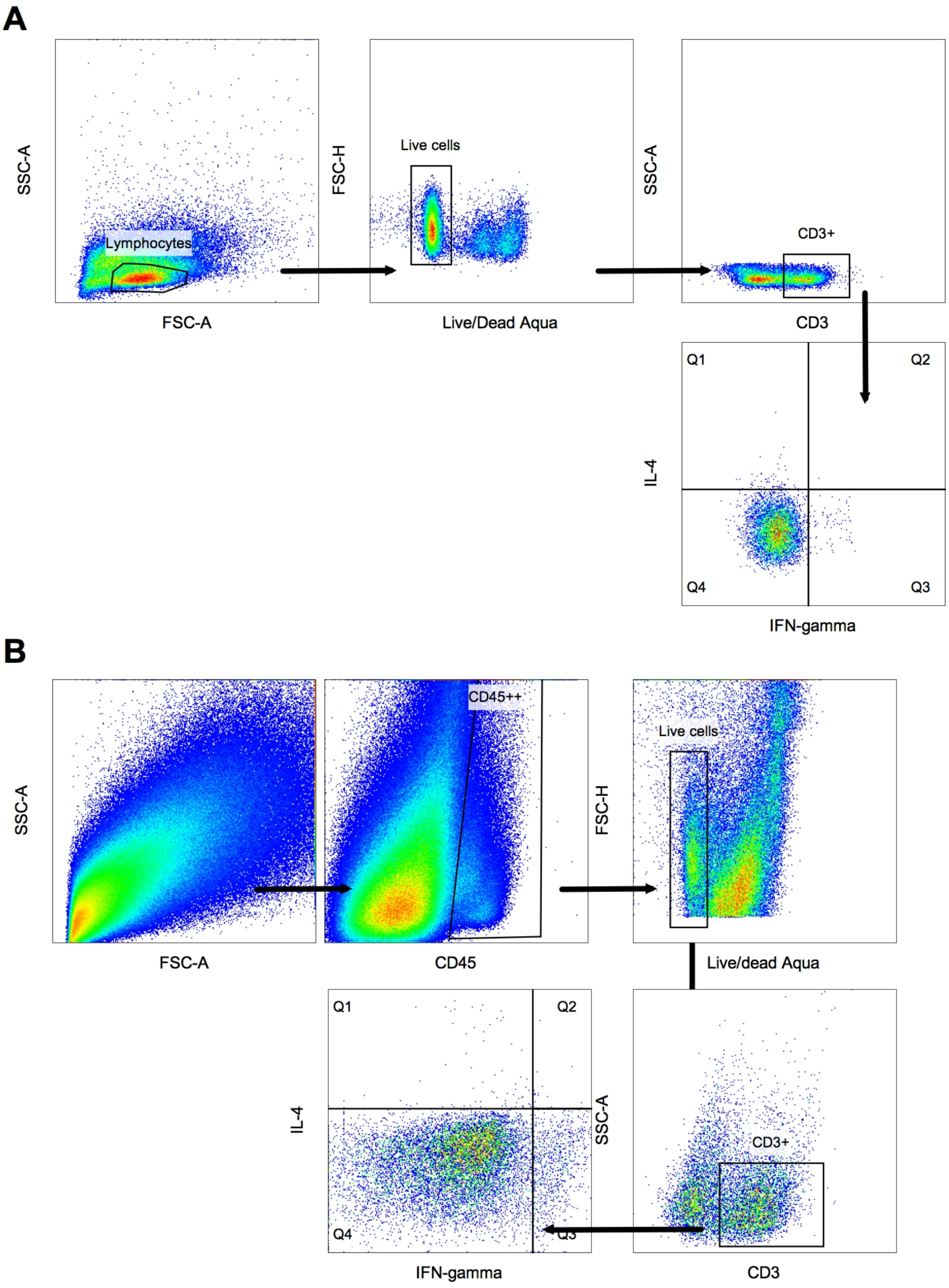

Supplementary Figure S4. Representative gating strategy for **(a)** circulating and **(b)** renal IFN-γ- and IL-4-producing T cells. For circulating T cells, lymphocytes were gated based on forward and side scatter properties, from which viable cells and subsequently CD3+ cells were gated. For renal T cells, CD45+ cells were gated from the original forward scatter and side scatter plot, Viable cells were then gated from which CD3+ cells were isolated. IFN-γ- and IL-4-producing T cells were gated based on an unstimulated blood and renal samples.

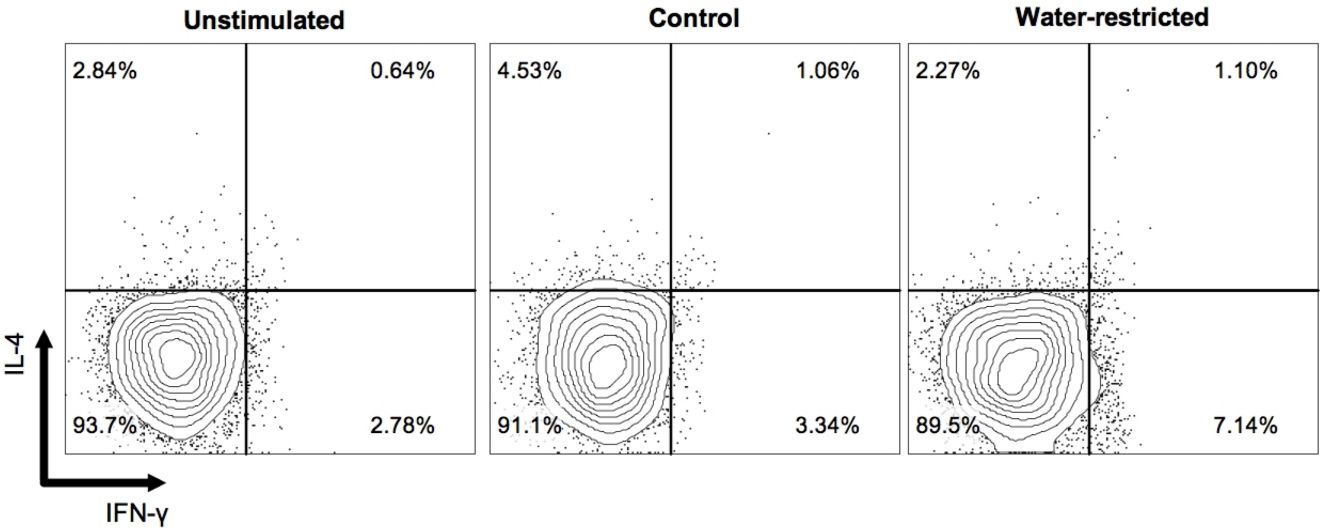

Supplementary Figure S5. Representative flow cytometric plots of gating for IFN-γ+ and IL-4+ cells from unstimulated renal T cells (left), and PMA-ionomycin-stimulated renal T cells from control (center) and water-restricted (right) SHR.
